# Search for compound heterozygous effects in exome sequence of unrelated subjects

**DOI:** 10.1186/1753-6561-5-S9-S95

**Published:** 2011-11-29

**Authors:** G Bryce Christensen, Christophe G Lambert

**Affiliations:** 1 Golden Helix Incorporated, 203 Enterprise Boulevard, Suite 1, Bozeman, MT 59718, USA

## Abstract

To enable the assessment of compound heterozygosity, we propose a simple approach for incorporating genotype phase in a rare variant collapsing procedure for the analysis of DNA sequence data. When multiple variants are identified within a gene, knowing the phase of each variant may provide additional statistical power to detect associations with phenotypes that follow a recessive or additive inheritance pattern. We begin by phasing all marker data; then, we collapse nonsynonymous single-nucleotide polymorphisms within genes on each phased haplotype, resulting in a single diploid genotype for each gene, which represents whether one or both haplotypes carry a nonsynonymous variant allele. A recessive or additive association test can then be used to assess the relationship between the collapsed genotype and the phenotype of interest. We apply this approach to the unrelated individuals data from Genetic Analysis Workshop 17 and compare the results of the additive test with a dominant test in which phase is not informative. Analysis of the first phenotype replicate shows that the *FLT1* gene is significantly associated with both Q1 and the binary affection status phenotype. This association was detected by both the additive and dominant tests, although the additive phase-informed test resulted in a smaller *p*-value. No false-positive results were detected in the first phenotype replicate. Analysis of the average values of all phenotype replicates correctly identified five other genes important to the simulation, but with an increase in false-positive rates. The accuracy of our method is contingent on correct phase determination.

## Background

Modern high-throughput DNA sequencing technology enables the detection of genetic variants at the most fundamental level. To analyze sequence data across large cohorts of individuals, investigators need to develop and implement methods that will intelligently combine the information from rare genetic variants that may share functional relationships. Genetic Analysis Workshop 17 (GAW17) provides an excellent opportunity to test such analysis methods in the context of an exome sequencing project [1]. The GAW17 data are based on sequence data from the 1000 Genomes Project pilot3 study and simulated phenotypes. The data consist of 697 subjects from seven ethnic groups representing African, Asian, and European populations. Exome data are particularly interesting because exonic sequence variants have a high probability of influencing the function of genes. Analysis of whole-exome sequence data has already proved to be effective for identifying the genetic cause of several diseases, particularly for rare recessive diseases.

A common method for analyzing rare sequence variants is to pool or collapse all the variants that occur in a defined region (e.g., a gene, an exon, or a defined moving window) and to test for the presence or absence of any sequence variants within the region [2]. This approach can be extended to consider only certain types of variants, such as single-nucleotide polymorphisms (SNPs) that result in nonsynonymous amino acid changes or SNPs that are not characterized in catalogs of genomic variants. The practice of collapsing rare variants assumes that rare sequence variants occurring within a defined region are functionally similar and can therefore be considered a single allele, but current collapsing procedures do not consider the haplotypic phase of the variants, which is important for recessive and additive traits [2]. Autosomal recessive phenotypes are expressed when an individual inherits two copies of a variant allele. The probability of an additive phenotype being expressed increases with the number of variant alleles.

The condition of inheriting DNA sequence variants at two sites within a gene, with each variant coming from a different parent, is called compound heterozygosity. Each locus is heterozygous when considered individually, but the compound effect of having variants in each of the two copies of an autosomal gene may be functionally analogous to a homozygous mutation. In a family-based study, Pierce et al. [3] identified compound heterozygosity as the cause of a rare recessive disease based on their analysis of exome sequence data in which phase could be accurately inferred.

This finding leads us to ask whether it is possible to identify similar phenomena in a cohort of unrelated subjects. We propose extending the process of rare variant collapsing to combine the information from variants occurring together on phased haplotypes in order to utilize compound heterozygosity to inform association tests in a population-based cohort. We present this analysis of the GAW17 exome sequence data as a proof of concept. The major obstacle to successful detection of compound heterozygotes in unrelated individuals is the difficulty in determining genotype phase for rare genetic variants. The genetic variants found by sequencing may be rare or even unique to a single individual. Genotype phase for the unrelated subjects in the GAW17 data must be inferred from linkage disequilibrium patterns, which are difficult to assess accurately for rare variants. Algorithmic phase estimation might introduce artifacts in the analysis process. We therefore present results using two different phasing algorithms to assess the robustness of the proposed method. The results obtained from the phase-informed analyses are compared to the results obtained using a similar analysis method that does not consider phase.

## Methods

We determined genotype phase for all SNPs in the GAW17 exome sequence data using both fastPHASE [4] and Beagle [5]. The GAW17 data were distributed as phased genotypes with phasing determined by fastPHASE [1]. Beagle was run with the options “nsamples=10” and “niterations=10.” We also determined a single compound genotype for each gene in each of the two phased data sets, using the letter *A* to indicate the absence of a nonsynonymous variant and the letter *B* to indicate the presence of such a variant. The genotype was therefore homozygous for the variant allele (*B*/*B*) if at least one nonsynonymous variant was found on each haplotype in the gene, heterozygous (*B*/*A*) if nonsynonymous variants were identified on only one haplotype, and homozygous for the common or reference allele (*A*/*A*) if no nonsynonymous variants were found in the gene. Mutation type (synonymous or nonsynonymous) and the variant allele for each SNP were determined from the annotations distributed with the raw data [1].

The compound genotype can be tested using any standard statistic for genotype associations. The collapsing procedure identified only a small number of subject-gene combinations with nonsynonymous variants on both haplotypes (equating to genotype *B*/*B*), making a recessive test uninformative. We therefore chose to concentrate on an additive model, for which the limited number of *B*/*B* genotypes can still provide additional information over methods that do not consider phase. We used logistic regression to test for association of the primary phenotype (Affected) with the compound genotypes produced by each phasing method using an additive inheritance model. We ran the logistic regression again with a dominant inheritance model to assess the significance of collapsing rare variants within genes without phasing. The dominant model tests for the presence of one or more nonsynonymous variants in the gene, regardless of phase. We used linear regression to test for associations with the three quantitative traits (Q1, Q2, and Q4), again using additive models for the compound genotypes from each phasing method as well as a dominant model. The analysis concentrated on the first phenotype simulation replicate using the entire cohort of 697 subjects.

All tests were adjusted for population stratification using principal components analysis (PCA). We calculated principal components using a subset of 4,360 SNPs with minor allele frequency (MAF) greater than 0.01 and maximum pairwise linkage disequilibrium of *R*^2^ = 0.5. The first three principal components were included as covariates in the regression analyses. All statistical tests, including PCA, were performed with the Golden Helix SNP and Variation Suite (SVS), version 7.4.0-Beta [6]. Data processing was performed using both SVS and R [7]. We designed and executed our analysis without knowledge of the underlying phenotype simulation parameters. Informal comparisons between standard and phase-informed analysis methods are made based on the total number of false-positive findings and the number of simulated trait loci correctly identified at the specified significance level.

## Results

We performed tests on 2,196 genes. The Bonferroni significance threshold for this number of tests is 2.27 × 10^−5^. A summary of results is shown in Table 1. The *FLT1* gene on chromosome 13 was significantly associated in all three tests for the binary phenotype (affected status). Tests based on the Beagle phasing method identified four case subjects and two control subjects as *FLT1* compound heterozygotes. The fastPHASE method identified three case subjects and no control subjects as *FLT1* compound heterozygotes (see Table 2). This small imbalance in compound heterozygotes between case and control subjects resulted in lower *p*-values for the additive tests (*p* = 2.21 × 10^−6^, *p* = 1.04 × 10^−6^) than for the dominant test (*p* = 3.75 × 10^−6^). Association testing for Q1 also showed an extremely strong association at *FLT1*. Based on these results, we believe that *FLT1* is an important factor for Q1 and the binary affection phenotype. No other genes reached the prescribed significance threshold for any phenotype based on the first phenotype simulation replicate. The strongest statistical association for Q2 was found at *VNN1* (*p* = 2.32 × 10^−4^, Beagle method). The strongest association for Q4 was found at *GRIA4* (*p* = 1.18 × 10^−4^, Beagle method).

**Table 1:** Results of association testing on the first phenotype replicate

	Additive model, Beagle		Additive model, fastPHASE		Dominant model (no phase)	
	
Phenotype	Gene	*p*-value	Gene	*p*-value	Gene	*p*-value
Affected	FLT1*	2.21 × 10^−6^	FLT1*	1.04 × 10^−6^	FLT1*	3.75 × 10^−6^
	*GRK4*	1.25 × 10^−4^	*GRK4*	1.49 × 10^−4^	*AHSA2*	2.30 × 10^−4^
	*AHSA2*	2.30 × 10^−4^	*AHSA2*	2.30 × 10^−4^	*B4GALT6*	4.64 × 10^−4^
Q1	FLT1*	1.70 × 10^−20^	FLT1*	7.24 × 10^−20^	FLT1*	3.73 × 10^−18^
	*ZNF550*	3.63 × 10^−4^	*ZNF550*	3.63 × 10^−4^	*ZNF502*	1.37 × 10^−4^
	*KIAA1542*	7.05 × 10^−4^	*KIAA1542*	7.15 × 10^−4^	*HNRPUL1*	1.03 × 10^−3^
Q2	VNN1*	2.32 × 10^−4^	*SDPR*	3.84 × 10^−4^	*KRT9*	8.67 × 10^−4^
	*SDPR*	3.84 × 10^−4^	VNN1*	4.06 × 10^−4^	*SDPR*	8.87 × 10^−4^
	*RUNX3*	4.97 × 10^−4^	*RUNX3*	4.97 × 10^−4^	*TRPV6*	1.17 × 10^−3^
Q4	*GRIA4*	1.18 × 10^−4^	*GRIA4*	4.35 × 10^−4^	*GRIA4*	1.18 × 10^−4^
	*ABL1*	2.81 × 10^−4^	*ABL1*	2.81 × 10^−4^	*ABL1*	2.81 × 10^−4^
	*GRK4*	4.16 × 10^−4^	*ICAM4*	4.36 × 10^−4^	*SLC22A1*	4.00 × 10^−4^

The three genes with the smallest *p*-values are listed for each test. Genes involved in the GAW17 phenotype simulation are marked with an asterisk.

**Table 2:** Genotype counts for selected genes

Gene	Method	Group	*A*/*A*	*B*/*A*	*B*/*B*
*FLT1*	Beagle	Case	142	63	4
		Control	412	74	2
	fastPHASE	Case	142	64	3
		Control	412	76	0
*VNN1*	Beagle	Case	136	58	15
		Control	348	123	17
	fastPHASE	Case	136	59	14
		Control	348	123	17

*A*/*A* indicates that no nonsynonymous variants are present, *B*/*A* indicates that nonsynonymous variants were observed on only one haplotype, and *B*/*B* indicates that nonsynonymous variants were observed on both haplotypes. Counts are based on two phasing methods, Beagle and fastPHASE.

We repeated the additive association tests using the average of each phenotype across the 200 simulation replicates, with the assumption that the averaged phenotypes would give an accurate representation of the simulation parameters and the best estimate of each subject’s disease liability. Increased phenotypic accuracy should improve the power of the tests and reduce the stochastic noise inherent in analyzing a single simulation replicate. Tests were performed for the mean of the 200 simulated values for the quantitative traits. For the binary affection status, we counted the number of times each subject was affected in the 200 replicates and used this count as a quantitative response variable. A summary of the results from these tests is shown in Table 3. Table 4 contains a list of all true-positive and false-positive associations identified with each analysis approach. Findings are generally similar for the various analysis approaches, with the notable difference that both of the phased approaches correctly identify the *KDR* gene as associated with Q1, whereas the unphased approach did not find this gene.

**Table 3:** Results of association testing for averaged phenotypes across all 200 simulation replicates

	Additive model, Beagle		Additive model, fastPHASE		Dominant model (no phase)	
	
Phenotype	Gene	*p*-value	Gene	*p*-value	Gene	*p*-value
Affected	FLT1*	4.70 × 10^−8^	FLT1*	1.08 × 10^−7^	FLT1*	2.90 × 10^−7^
	PIK3C2B*	3.75 × 10^−5^	*HPDL*	5.44 × 10^−5^	*HPDL*	5.44 × 10^−5^
	*HPDL*	5.44 × 10^−5^	*GRIA4*	9.43 × 10^−5^	*GRIA4*	9.43 × 10^−5^
Q1	FLT1*	7.43 × 10^−61^	FLT1*	9.61 × 10^−57^	FLT1*	2.04 × 10^−58^
	*SLC2A13*	7.22 × 10^−8^	*SLC2A13*	3.02 × 10^−7^	*SLC2A13*	7.22 × 10^−8^
	KDR*	1.45 × 10^−6^	KDR*	1.12 × 10^−6^	*PSKH2*	2.26 × 10^−6^
Q2	VNN1*	1.98 × 10^−16^	VNN1*	9.42 × 10^−17^	VNN1*	5.89 × 10^−13^
	RARB*	7.64 × 10^−7^	RARB*	7.64 × 10^−7^	RARB*	7.64 × 10^−7^
	*TXNL1*	1.26 × 10^−6^	*TXNL1*	1.25 × 10^−6^	*SIRT1*	7.95 × 10^−7^
Q4	*ICAM4*	3.35 × 10^−4^	*GLP2R*	1.38 × 10^−4^	*LP2R*	6.22 × 10^−5^
	*GLP2R*	3.62 × 10^−4^	*GOLGA1*	2.25 × 10^−4^	*YP3A43*	1.11 × 10^−4^
	*PBX3*	5.79 × 10^−4^	*ICAM4*	3.35 × 10^−4^	*LC22A1*	2.22 × 10^−4^

Tests were run using the average phenotype values from the 200 simulation replicates. The three genes with the smallest *p*-values are listed for each test. Genes involved in the GAW17 phenotype simulation are marked with an asterisk.

**Table 4:** Genes found by each analysis approach

	Analysis of first simulation replicate			Analysis of average values of all 200 simulation replicates		
	
Trait	Additive model, Beagle	Additive model, fastPHASE	Dominant model (no phase)	Additive model, Beagle	Additive model, fastPHASE	Dominant model (no phase)
Affected	FLT1*	FLT1*	FLT1*	FLT1*	FLT1*	FLT1*
Q1	FLT1*	FLT1*	FLT1*	FLT1*	FLT1*	FLT1*
				KDR*	KDR*	*SLC2A13*
				*SLC2A13*	*SLC2A13*	*PSKH2*
				*MAP3K12*	*MAP3K12*	*LIMK2*
				*PATE*	*PATE*	*JAK1*
				*JAK1*	*JAK1*	
Q2	None	None	None	VNN1*	VNN1*	VNN1*
				RARB*	RARB*	RARB*
				SIRT1*	SIRT1*	SIRT1*
				GCKR*	GCKR*	GCKR*
				*TXNL1*	*TXNL1*	*TXNL1*
				*OR5B2*	*OR5B2*	*OR5B2*
				*MAF*	*MAF*	*MAF*
				*PCDHGB2*	*PCDHGB2*	*PCDHGB2*
				*C3ORF30*	*C3ORF30*	
				*TRPV6*		
Q4	None	None	None	None	None	None

Given the Bonferroni significance threshold for 2,196 tests (*p* < 2.27 × 10^−5^), we show the genes identified as significant for each phenotype using each analysis approach. Genes involved in the GAW17 phenotype simulation are marked with an asterisk.

## Discussion and conclusions

The intent of this analysis was to assess the feasibility of incorporating compound heterozygosity into an association test based on exome sequence data with unrelated subjects. Our approach used a simple method to collapse nonsynonymous variants on phased haplotypes within genes, and we compared results of an additive model incorporating compound heterozygosity with an analogous dominant model for which phase was not informative. We used two phasing methods to ensure that our approach was robust to phase estimation artifacts and found the results to be fairly consistent for the most significant genes. It is important to note that phase information was not included in the phenotype simulation model. This analysis is only a proof of concept. The additive tests using phase information resulted in lower *p*-values for some genes, but not all. The true phase of the SNPs in these data is not known, but if we assume that our phasing methods are accurate, our results show that additional power can be gained in some situations by incorporating compound heterozygosity in a large-scale analysis of sequence data.

Although it is discouraging that we detected only one true positive association in the first phenotypic replicate, it is also encouraging that there were no false-positive associations. (We should note that we found numerous false-positive associations in the preliminary results before the PCA correction was incorporated. For example, the uncorrected analysis for Q1 resulted in false-positive associations with five genes in addition to the one true-positive association with *FLT1*.) As expected, analysis of the averaged phenotypes resulted in greater statistical power but also in an increase in false positives. No false associations were detected for Q4 or the binary affection trait, but there were numerous false positives for Q1 and Q2. The false-positive rates were similar for the phased and unphased approaches.

We believe that the major weakness of our method lies in the difficulty of inferring genotype phase for rare SNPs. Most of the nonsynonymous variants in the data were very rare. Out of 13,572 total nonsynonymous SNP variants in the data, 5,924 occurred only once and another 1,505 occurred only twice. The greatest certainty for phase determination is in the genes with numerous and relatively common variants. Genes with numerous variants also have additional statistical power to detect associations using the collapsing approach that we followed. As such, it is not surprising that most of the genes we identified as being associated harbor multiple variants with relatively high frequencies. Although the two phasing methods we used generally resulted in similar compound genotypes (Table 2) and similar statistically significant findings, there were numerous inconsistencies, especially with regard to the rarest SNPs. We identified 119 instances of a subject having exactly two nonsynonymous personal variants (variants unique to that subject’s sequence) within one gene. Beagle determined that for 68 of these 119 subjects, the variants were on opposite haplotypes, resulting in a compound heterozygote. In contrast, FastPHASE resulted in compound heterozygote genotypes for only 51 subjects.

In practice, we expect that our method will have the greatest value when applied to low-frequency variants. High-frequency variants might confound the collapsing process and hide the influence of rare deleterious variants. We expect sequencing data sets to remain small in the near future, which means that inferring phase from observed linkage disequilibrium patterns will be especially difficult for common and rare variants alike. We hope that future advances in sequencing technology will make it possible to assess phase more accurately or even to observe phase directly. This could be made possible by single-molecule analysis, extended read lengths, or advanced sequence capture technology. As DNA sequencing technology continues to improve, we anticipate that the methods we describe here will become useful for analysis of sequence data in large cohorts of unrelated subjects. Our method may also be adapted for use in analysis of rare SNPs assayed by modern array-based SNP genotyping platforms.

## Competing interests

The authors are employees of Golden Helix, Inc., a commercial software provider whose “SNP & Variation Suite” software was used extensively in this analysis.

## Authors’ contributions

GBC conceived of the study, performed all analytical work and drafted the manuscript. CGL contributed to the study design and helped to draft the manuscript. All authors read and approved the final manuscript.
